# Supplementary material for: Evoked transients of pH-sensitive fluorescent false neurotransmitter reveal dopamine hot spots in the globus pallidus
Source: eLife. 2018 Dec 19;7:e42383. doi: 10.7554/eLife.42383 (PMC6324876; doi:10.7554/eLife.42383)
Supplement: Transparent reporting form [file elife-42383-transrepform.pdf]

eLife's transparent reporting form

We encourage authors to provide detailed information *within their submission* to facilitate the interpretation and replication of experiments. If you have any questions, please contact us: editorial@elifesciences.org.

Sample-size estimation

- You should state whether an appropriate sample size was computed when the study was being designed
- You should state the statistical method of sample size computation and any required assumptions
- If no explicit power analysis was used, you should describe how you decided what sample (replicate) size (number) to use

Please outline where this information can be found within the submission (e.g., page numbers or figure legends), or explain why this information doesn't apply to your submission:

The optical method we use in this study to quantitatively measure dopamine synapse activity is a novel approach and no studies have estimated effect sizes for any manipulations. We calibrated our sample size based on the differences we saw between FFN treated slices and untreated slices. With $\alpha=0.01$, we observed a significant difference with n=6 slices (Fig 2), which we therefore selected as a minimum number of slices for comparing between treated and untreated slices where the unit of analysis is a slice. For analysis of more subtle relationships, such as calcium and dependence on reuptake, we relied on larger sample sizes (Fig 3, Fig 5, and Fig 6). For exploratory analysis, we treated every field of view as an independent unit and increased our sampling to obtain sufficient data for drawing conclusions about a range of image characteristics (captured in the spread of Canny Edge sum values, Fig 7).

Replicates

- You should report how often each experiment was performed
- You should include a definition of biological versus technical replication
- The data obtained should be provided and sufficient information should be provided to indicate the number of independent biological and/or technical replicates
- If you encountered any outliers, you should describe how these were handled
- Criteria for exclusion/inclusion of data should be clearly stated
- High-throughput sequence data should be uploaded before submission, with a private link for reviewers provided (these are available from both GEO and ArrayExpress)

Please outline where this information can be found within the submission (e.g., page numbers or figure legends), or explain why this information doesn't apply to your submission:

Experiments involved collecting data from many animals, with each animal yielding between three or four slices. For experiments where the unit of analysis was the animal or the slice, including measuring calcium dependence (Fig 3) and functional anatomy (Fig 4), animals were sacrificed and imaged daily or every other day, for as many days as required to obtain data. For a larger data set where the unit of analysis was field of view (Fig 7), data was collected from mice of similar ages over a short period of several weeks to minimize the influence of confounding factors.

These experiments are not designed as "replicates", as each time the experiment was run, it is another "n" or potentially multiple "n" (depending on the unit of analysis) included in the total report of the data. This is typical for physiology experiments, as each experimental subject, in this case each mouse brain, is independent.

Statistical reporting

- Statistical analysis methods should be described and justified
- Raw data should be presented in figures whenever informative to do so (typically when N per group is less than 10)
- For each experiment, you should identify the statistical tests used, exact values of N, definitions of center, methods of multiple test correction, and dispersion and precision measures (e.g., mean, median, SD, SEM, confidence intervals; and, for the major substantive results, a measure of effect size (e.g., Pearson's r, Cohen's d)
- Report exact p-values wherever possible alongside the summary statistics and 95% confidence intervals. These should be reported for all key questions and not only when the p-value is less than 0.05.

Please outline where this information can be found within the submission (e.g., page numbers or figure legends), or explain why this information doesn't apply to your submission:

(For large datasets, or papers with a very large number of statistical tests, you may upload a single table file with tests, Ns, etc., with reference to page numbers in the manuscript.)

Figure legends contain this information. Additionally, readers can download and run the MATLAB data and annotated source code for Figures 3,5,6, and 7.

Additional data files ("source data")

- We encourage you to upload relevant additional data files, such as numerical data that are represented as a graph in a figure, or as a summary table
- Where provided, these should be in the most useful format, and they can be uploaded as "Source data" files linked to a main figure or table
- Include model definition files including the full list of parameters used
- Include code used for data analysis (e.g., R, MatLab)
- Avoid stating that data files are "available upon request"

Please indicate the figures or tables for which source data files have been provided:

For figures presenting summaries of larger datasets, we have uploaded MATLAB data and annotated source code. These are Figures 3,5,6, and 7.
